# Pancreatic Cancer: Beyond Brca Mutations

**DOI:** 10.3390/jpm12122076

**Published:** 2022-12-16

**Authors:** Vincenzo Ricci, Teresa Fabozzi, Maria Anna Bareschino, Emiddio Barletta, Domenico Germano, Immacolata Paciolla, Vincenza Tinessa, Antonio Maria Grimaldi

**Affiliations:** 1 Medical Oncology Unit, AORN “San Pio”, 82100 Benevento, Italy; 2 Medical Oncology Unit, Ospedale del Mare, 80147 Naples, Italy

**Keywords:** metastatic pancreatic cancer, target therapy, pathway, PARP inhibitor, BRCA mutation

## Abstract

Pancreatic cancer is the fourth-leading cause of cancer-related deaths worldwide. The outcomes in patients with pancreatic cancer remain unsatisfactory. In the current review, we summarize the genetic and epigenetic architecture of metastatic pancreatic cancer beyond the BRCA mutations, focusing on the genetic alterations and the molecular pathology in pancreatic cancer. This review focuses on the molecular targets for the treatment of pancreatic cancer, with a correlation to future treatments. The potential approach addressed in this review may lead to the identification of a subset of patients with specific biological behaviors and treatment responses.

## 1. Introduction

Pancreatic ductal adenocarcinoma (PDAC) is one of the deadliest malignancies, with a 5-year overall survival rate of less than 10% [1]. Although most cases of PDAC are considered sporadic, 5–10% are familial pancreatic cancer (FPC) [2].

Surgery is the main treatment for PDAC, but it is often diagnosed in advanced disease and surgical resection is not possible. Only 15–20% of PDAC cases are resectable, and over 75% of them have a disease recurrence within 5 years after the surgical resection [3]. Gemcitabine has been implicated in the treatment of inoperable pancreatic cancer, with low response rates and a modest overall survival benefit.

Combination chemotherapy with FOLFIRINOX is more effective than gemcitabine alone. Albumin-bound paclitaxel (nabpaclitaxel) can also be used in combination with gemcitabine for the treatment of pancreatic cancer. In the first-line setting, both have been shown to prolong survival, compared to gemcitabine monotherapy. Even with these treatments, the 2-year survival rate remains at 10%, with an overall median survival (OS) ranging from 8 to 11 months. Second-line chemotherapy may include FOLFIRINOX, FOLFOX, or FOLFIRI regimens. Monochemotherapy with gemcitabine, capecitabine, or 5-fluorouracil is preferred for patients with a poor performance status [4].

Effective treatments for pancreatic cancer need to be developed, as these treatments prolong survival. Recently, genomic analysis of PDAC identified KRAS, CDKN2A, TP53, and SMAD4 as common and somatically mutated genes [5,6]. Mutations in the genes involved in the breast cancer susceptibility gene (BRCA) signaling pathway, namely BRCA1, BRCA2, and PALB2, have been found in a subset of PDAC. BRCA1 and BRCA2 genes are part of the homologous DNA repair pathway (HR), and the mutations in these genes are powerful contributors to the development of several cancers, including breast, prostate, ovarian, and pancreatic cancers [7]. The BRCA pathway-mutant PDACs are defective in DNA double-strand break repair, and may respond better to platinum-based chemotherapy or poly ADP-ribose polymerase (PARP) inhibitors.

Therefore, studies on the molecular epidemiological and clinicopathological characteristics of the BRCA signaling pathway-mutant PDACs are important for implementing therapeutic strategies for pancreatic cancer [8].

## 2. Materials and Methods

The selected references were identified by electronic searches in PubMed, Medline, and Web of Science using the following words: “pancreatic cancer”, “BRCA1”, “BRCA2”, “angiogenesis”, “EGFR’’, “IGF1R”, “RAS”, “PI3K”, ‘‘Akt”, “mTOR”, “Src”, “JAK/STAT”, “Notch”, “Hedgehog”, “Wnt”, and “TGF-β”. The last search was updated on 21 July 2022. This work used MeSH terms and free-text words to enhance the search sensitivity. This review was conducted according to the standard guidelines for systematic reviews [9].

### 2.1. Inclusion and Exclusion Criteria

The authors independently reviewed all the eligible studies according to the following criteria: (1) the response to the pancreatic cancer therapy; (2) the genetic mutations and molecular pathology of the pancreatic cancer; (3) the BRCA1/2 mutations and therapeutic regulation; and (4) the publishing of the full text in English. The exclusion criteria were: (1) abstracts; (2) the studies not including pancreatic cancer response; (3) the studies with no available data; (4) the research published in a language other than English; and (5) duplicate releases.

### 2.2. Data Extraction

The authors independently reviewed and extracted data from all the identified studies based on the inclusion and exclusion criteria.

## 3. Results

### 3.1. GEne Alteration and Molecular Pathology of Pancreatic Cancer

Pancreatic cancer has an average of 63 gene mutations. The molecular analysis of pancreatic cancer often reveals the role of the known oncogenes and the cancer signaling pathways. The mutated oncogene KRAS, found in advanced pancreatic cancer, encodes a small GTPase that regulates the downstream signaling from growth factor receptors [10,11].

The KRAS mutations are involved in the progression of human pancreatic intraepithelial neoplasia (PanIN) [12,13]. Other mutations in the tumor-suppressor genes, such as INK4A, LKB1, and BRCA2, are common.

The onco-suppressor gene P16/CDKN2A is inactivated in more than 90% of pancreatic cancer cases [14]. The mutations in the p53 gene are strongly associated with the cellular responses to cytotoxic stress and contribute to apoptosis and cell cycle arrest [15].

Additionally, the mutations in p53 are commonly found in approximately 75% of pancreatic cancer patients. The tumor-suppressor genes represented by the missense mutations in SMAD4, which encodes the transforming growth factor beta (TGFβ) signaling, are found in about 55% of pancreatic cancer patients, and are associated with a poor prognosis [16]. In addition, the mismatch repair gene MLH1 and the cationic trypsinogen gene (PRSS1) are also frequently mutated. These mutations are thought to influence the malignant progression [17]. Some pancreatic cancers have activating mutations in BRAF but not in KRAS [18]. BRAF encodes RAF, a serine/threonine kinase belonging to the MEK family. MEK activates ERK, forming the MAPK signaling pathway. Active mutations in KRAS and BRAF, therefore, ultimately lead to the triggering of the MAPK signaling pathway, which is critical for the pancreatic cancer development [19]. The phosphoinositide-3-kinase (PI3K) signaling is another important pathway, in addition to the MAPK pathway.

The PI3K pathway promotes survival and cell proliferation through several molecules, such as Akt, p70-S6K, and mTOR. The PI3K signaling, which is enhanced in 10% to 20% of pancreatic cancers, has been associated with pancreatic cancer oncogenesis [20]. The vascular endothelial growth factor (VEGF) and the insulin-like growth factor-1 receptor (IGF1R) are pathways abnormally expressed and involved in cellular functions, such as apoptosis, spread, and metastasis [21], see Table 1.

### 3.2. Angiogenesis

Angiogenesis is important for tumor growth and spread [22].

VEGF plays an important role in angiogenesis, which favors proliferation and metastasis [23].

VEGF is overexpressed in more than 90% of metastatic pancreatic ductal adenocarcinomas (mPDACs), which explains the role of targeted VEGF therapy [24].

In a large, randomized phase III trial (CALGB 80303), no improvement was found in the OS in the patients receiving the bevacizumab (a monoclonal antibody directed against VEGF) and the gemcitabine, compared to the outcomes in patients receiving the gemcitabine and the placebo, despite promising results in the phase II clinical trial [25].

The multi-targeted kinase inhibitors sorafenib and axitinib have proven ineffective [26,27].

Foretinib is being developed as an ATP-binding site competitor that inhibits receptor tyrosine kinases with reported activity against the VEGFR, c-Met, RON, FLT-3, c-KIT, and platelet growth factor receptor (PDGFR). Multiple lines of evidence suggest that foretinib targets several additional kinases and inhibits tumor growth [28].

The c-MET and Hepatocyte growth factor (HGF) are often overexpressed [29,30].

### 3.3. EGFR

The epidermal growth factor receptor (EGFR) plays a crucial role in tumor cell activity.

The abnormal EGFR activity causes receptor dimerization, which then activates the downstream signaling involving the components of the RAS and the PI3K/Akt/mTOR signaling [31]. The EGFR inhibitors cause a decreased proliferation in the pancreatic cancer cell lines [32].

The clinical trials with anti-EGFR and anti-ErbB2 antibodies have not demonstrated positive results [33,34].

In a randomized phase III study that assigned patients with mPDAC to treatment with gemcitabine with or without erlotinib, the patients who received the combination treatment showed an improved overall survival (OS) (*p* = 0.038) and progression-free survival (PFS) (*p* = 0.004). The subgroup analysis did not indicate whether the KRAS mutational status or EGFR were predictive markers of the treatment response to erlotinib.

Although the median OS was only extended by 2 weeks, this study is important because it is the only one to show an improved OS with the erlotinib/gemcitabine combination in mPDAC [35,36].

The anti-EGFR monoclonal antibody nimotuzumab achieved a longer survival with a tolerable toxicity when added to gemcitabine (8.7 months vs. 6.1 months), in a recent phase II trial in patients with locally advanced pancreatic cancer [37].

### 3.4. IGF1R

The IGF1R signaling, a member of the insulin receptor family, is abnormally expressed and its activation results in the signaling cascades which trigger signaling, such as ERK and PI3K/Akt/mTOR.

It is involved in cancer survival and utilized in the RAS pathway.

In a previous study, the IGF1R inhibitor AMG-479 and the monoclonal antibody cixutumumab did not improve survival [38]. Another study showed that concurrent blockade of IGF1R and its EGFR/Her-2 simultaneously inhibited the pancreatic tumor growth and the stimulation of IRS-1, Akt, and MAPK phosphorylation. Therefore, the combination of these two inhibitors overcomes the resistance associated with a single agent [39].

### 3.5. RAS

The RAS/RAF/MEK/ERK (MAPK) signaling is important in mediating growth and survival [40]. The MEK inhibitor trametinib has not demonstrated an improved OS when combined with gemcitabine in mPDAC [41]. A multicenter phase II study evaluated the safety and efficacy of the combination of the MEK1/2 inhibitors selumetinib and erlotinib in patients with previously treated advanced pancreatic cancer. The median PFS was 1.9 months (95% CI, 1.4–3.3 months), and the median OS was 7.3 months (95% CI, 5.2–8.0 months) [42].

### 3.6. PI3K/AKT/mTOR

PI3K activates Akt through the activation of RAS or EGFR, which in turn triggers multiple downstream targets, such as mTOR, affecting many cellular functions, including proliferation, apoptosis, spread, and chemoresistance [43].

The PI3K-Akt signaling is overexpressed in 59% of pancreatic cancer [44]. The downregulation of this signaling due to the reduced or absent expression of PTEN (a phosphatase and tensin homologue—a natural antagonist of PI3K) is common [45]. The mTOR inhibitor everolimus has demonstrated antitumoral activity, including the inhibition of tumor proliferation and angiogenesis [46]. A phase II trial evaluated the effect of association therapy with capecitabine and everolimus in patients with mPDAC, showing a response rate (RR) of 6.5% and an OS of 8.9 months; this demonstrates that this association may increase the response to chemotherapy [47].

### 3.7. SRC

Src, a family of non-receptor protein tyrosine kinases and overexpressed in 70% of pancreatic cancers, plays an important role in the regulation of multiple signaling pathways through interactions with the receptor tyrosine kinases and G protein-coupled receptors [48]. The Src kinase inhibitor dasatinib was evaluated in a phase II trial in patients with metastatic pancreatic cancer with a poor outcome [49]. A phase II study investigated the efficacy of dasatinib in combination with FOLFOX in mPDAC. The addition of dasatinib did not appear to confer additional clinical benefits to FOLFOX [50].

### 3.8. JAK/STAT

The overexpression of the Janus kinase/signal transducer and transcription pathway (JAK/STAT) was observed in several tumors [51]. The family of JAK kinases consists of four members: JAK1, JAK2, JAK3, and Tyk2. The JAK activation occurs when ligands bind to cell surface receptors, thereby creating sites for the interaction with proteins containing the phosphotyrosine-binding Src homology 2 (SH2) domains. STATs belong to the family of the transcription factors downstream of JAKs, containing tyrosine residues that are phosphorylated by JAKs [52]. The alterations in the JAK/STAT signaling pathways directly contribute to tumoral proliferation and angiogenesis [53,54]. A phase II trial of capecitabine, in combination with either the ruxolitinib or the placebo, improved the survival in patients with mPDAC [55].

### 3.9. Notch

Notch signaling is involved in tumor growth and survival, as well as in the development and function of many organs [56]. The NOTCH pathway regulates the pool of pancreatic progenitor cells early in the pancreatic development [57]. The Notch ligands favor the epithelial–mesenchymal transition (EMT) by the transcription factors, such as Slug, Snail, and TGFβ [58]. A phase II, randomized, placebo-controlled trial investigated the efficacy of tarectumab, a fully human IgG2 antibody that inhibits the Notch2/3 receptors, in combination with nab-paclitaxel and gemcitabine in patients with mPDAC. The addition of tarectumab to nab-paclitaxel and gemcitabine did not improve the OS, PFS, or overall response rate (ORR) [59]. A clinical trial is underway investigating the efficacy of the g-secretase inhibitor MK0752 in combination with gemcitabine in patients with mPDAC [60].

### 3.10. Hedgehog

The Hedgehog (HH) pathway regulates embryogenesis, with an increased expression seen during pancreatic carcinogenesis [61]. The Hedgehog signaling is found to be phosphorylated in early pancreatic tumors, and the activation of this signaling is important during the cellular progression and is associated with the KRAS mutations [62,63]. Moreover, the HH overexpression induces precancerous lesions in the transgenic mice and helps to maintain the tumor microenvironment [64]. The attenuation of the activity of the sonic HH resulted in the enhanced gemcitabine delivery and tumor angiogenesis in the mouse models, suggesting that this signaling may be an attractive target for new therapies [65]. In a phase II trial, the addition of vismodegib to the chemotherapy did not improve the efficacy compared to the chemotherapy alone in patients with mPDAC [66].

### 3.11. WNT

The Wnt pathway supports an important function in stem cell regulation, and recent studies have suggested its role in pancreatic cancer tumorigenesis. The dysregulation of the Wnt signaling may be closely associated with chemoresistance in pancreatic cancer [67,68,69]. A phase Ib trial evaluating vantictumab, a fully human monoclonal antibody that inhibits the Wnt pathway by binding to FZD1, 2, 5, 7, and 8 receptors, has been successful in patients with previously untreated metastatic pancreatic adenocarcinoma; the study is currently ongoing [70].

### 3.12. TGFβ

TGF-β is a key factor that plays a crucial role in many cellular processes, including cellular differentiation and epithelial–mesenchymal transition (EMT) [71]. Galunisertib is an oral small-molecule serine/threonine kinase inhibitor type I which converts the growth factor beta receptor (ALK5) [72]. The combination of galunisertib and gemcitabine improved the OS compared with the gemcitabine in patients with unresectable pancreatic cancer [73]. Vactosertib is an oral TGF-β inhibitor that targets the TGF-β type I receptor kinase. Two phase 1b trials are evaluating the safety of vactosertib, in combination with FOLFOX and nal-IRI/5FU/LV, in patients with metastatic pancreatic cancer who have failed first-line therapy with gemcitabine and nabpaclitaxel (NCT03666832-NCT04258072).

### 3.13. Stromal Environment

The extracellular matrix (ECM) and stromal cells are important barriers that limit the efficacy of the anticancer drugs [81]. The tumor–stroma interactions represent a complex signaling network that leads to tumor progression in many types of solid tumors. Pancreatic cancer is uniquely characterized by an abundant tumor stroma in the tumor microenvironment that impedes the passage of drugs into the tumor, and may induce complex interactions of cell–cell signaling. Stromal depletion strategies such as hyaluronic acid (HA) depletion may facilitate the drug delivery to tumor sites [82]. A phase III study evaluating the therapeutic activity of pegvorhyaluronidase alfa (PEGPH20), a pegylated recombinant hyaluronidase, in combination with nab-paclitaxel and gemcitabine (AG) in metastatic pancreatic cancer showed that the addition of PEGPH20 to AG increased the ORR, but it did not improve the OS and the PFS [83]. In a phase I/II trial comparing FOLFIRINOX with or without PEGPH20 in mPDAC patients, the addition of PEGPH20 to FOLFIRINOX appeared to increase the toxicity and be detrimental [84]. Heparan sulfate proteoglycans (HSPGs) are complex polysaccharides that promote tumorigenesis and are associated with the tumor microenvironment. M402, a heparan sulfate mimetic, inhibits multiple interactions related to heparan sulfate [85]. A phase II multicenter study (NCT01621243) evaluating the efficacy of M402 versus standard chemotherapy was closed because it showed an insufficient efficacy in the study population to warrant continuation.

### 3.14. Alternative Poly (Adp-RiboSE) Polymerase Pathway

**Figure 1:**
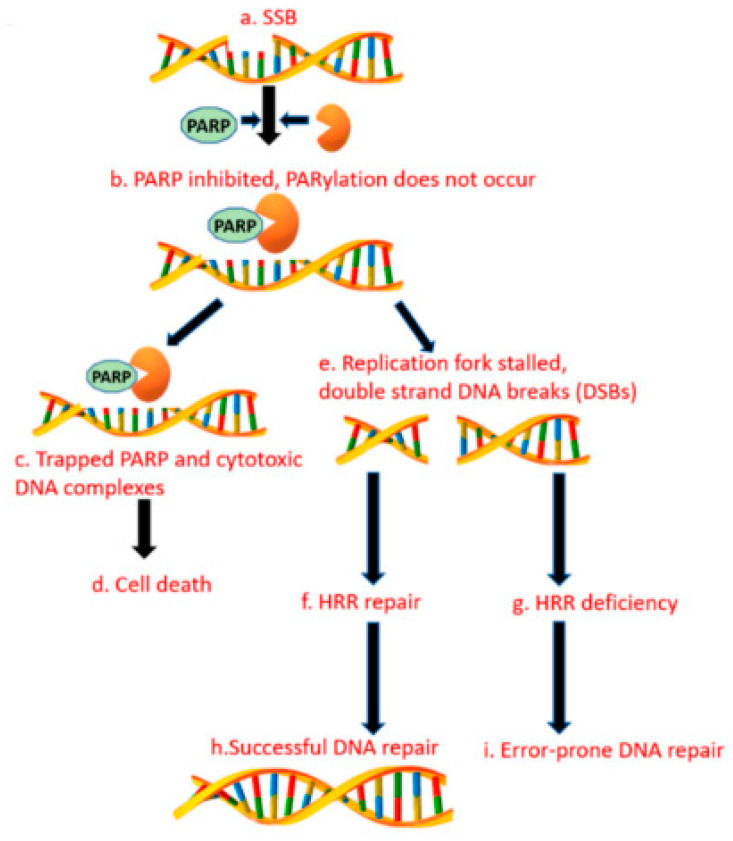
PARP enzyme inhibitors cause synthetic lethality in cancer cells with DNA repair defects or homologous repair defects.

Germline BRCA mutations (gBRCAms) are of increasing interest as treatments for BRCA-mutated ovarian and breast tumors. The gBRCA1/2 mutations increase the risk of pancreatic cancer and are found in up to 8% of sporadic pancreatic cancer patients. In patients with gBRCAms, the platinum-based chemotherapy and poly (ADP-ribose) polymerase inhibitors are effective treatments that prolong survival [74]. Several studies aimed at estimating the incidence of gBRCAms. The prevalence estimates ranged from 0.7% to 5.7% for BRCA2, and 0.3% to 2.3% for BRCA1 [75,76]. The BRCA1 mutations in FPC are less studied than the BRCA2. Zhen et al. demonstrated that gBRCA1 mutations were present in 1.2% of patients with the familial PDAC [77]. The DNA double-strand breaks are primarily repaired by homologous recombination (HR) mediated by the BRCA1 and BRCA2 proteins. The poly (ADP-ribose) polymerase pathway plays critical roles in the DNA repair when the BRCA dysfunction occurs [78]. The PARP proteins are involved in the cellular functions, including the DNA damage response, the DNA transcription, the maintenance of genomic stability, and the cell cycle regulation. The PARP enzyme inhibitors cause synthetic lethality in the cancer cells with the DNA repair defects or homologous repair defects. See Figure 1, these proteins have been shown to be clinically effective against cancers with the deficient DNA repair due to the germline mutations in the BRCA1 and BRCA2, which is estimated to account for 5% to 7% of pancreatic cancer patients. In a phase II study of cisplatin plus gemcitabine with or without veliparib, the PARP inhibitor (PARPi) did not improve the AE: Check formatting response rates in patients with mPDAC. These data establish cisplatin and gemcitabine as the standard of care for patients with the germline BRCA1/2 or PALB2 mutations (gBRCA/PALB2+) [79]. Another PARPi, olaparib, tested in a phase I trial at 100 mg b.i.d., in combination with Gemcitabine at 600 mg/m2, was well tolerated in patients with advanced pancreatic cancer [80]. The international, double-blind, placebo-controlled phase III POLO study evaluated the olaparib maintenance therapy in patients with mPDAC and gBRCA1/2 mutations who had not progressed during the first-line platinum-based chemotherapy. The patients were randomized to the maintenance therapy with the olaparib or the placebo. The olaparib maintenance showed statistically significant and clinically meaningful improvements in the PFS versus the chemotherapy (median 7.4 vs. 3.8 months; hazard ratio 0.53; 95% CI 0.35–0.82; *p* = 0.004) [86].

Golan T. et al. reported an update: the median OS was 19.2 months with the olaparib, and 19.0 months with the placebo (HR 0.83; 95% CI 0.56–1.22; *p* = 0.3487) but was not statistically significant [87].

## 4. Discussion

There are several unanswered questions regarding the POLO study. In particular, the unreached benefit in the OS supports the validity of the improved PFS. This may be due to the higher treatment rates in the placebo group after the disease progression, including 15% of patients who received PARPi. Because POLO only included patients with the gBRCA1/2 mutations, there were a larger number of mPDAC patients who may benefit from olaparib, including those with germline mutations in other components of the HR system (PALB2, ATM), or with different positive biomarkers for homologous recombination deficiency (HRD).

Rucaparib is another PARP inhibitor being tested for activity in clinical trials in breast and ovarian cancers with germline BRCA1/2 mutations. A phase II clinical trial of RUCAPANC enrolled 19 patients. In total, 16 of 19 of the BRCA1/2 mutations were germline and 3 were somatic. The patients received a median of two previous chemotherapy regimens. Four patients achieved responses, with two confirmed partial responses and one complete response (CR) (ORR 15.8%; 3 of 19), with an additional CR unconfirmed. The disease control rate was 31.6% [88].

A phase II, single-arm study evaluating the role of rucaparib as the maintenance therapy for advanced pancreatic cancer with the germline or somatic mutations in BRCA1, BRCA2, or PALB2 enrolled 46 patients, 42 of whom were eligible for the evaluation. The PFS rate at 6 months (PFS6) was 59.5% (95% CI 44.6 to 74.4), the median PFS was 13.1 months (95% CI 4.4 to 21.8), and the median OS was 23.5 months (95% CI 20 to 27) [89].

A new strategy for BRCA-mutant cancer is combination therapy with immune checkpoint inhibitors and PARPi [90]. Several studies have shown that BRCA2-mutant tumors have increased the susceptibility to immune checkpoint blockade due to its effects on the tumor immune microenvironment [91]. The addition of PARPi to the HRD cells determines the activation of the interferon gene-stimulating factor (STING) signaling pathway, which causes the cytosolic accumulation of DNA and an inflammatory cascade, thus leading to increased tumor infiltration by the lymphocytes and an increased PDL1 expression [92]. The randomized phase 1b/2 PARPVAX trial will enroll patients with pancreatic cancer who have not progressed on platinum chemotherapy, and they will receive niraparib plus either ipilimumab or nivolumab (NCT03404960). Another non-randomized phase 2 study, POLAR, is currently testing the combination of pembrolizumab and olaparib in patients with mPDAC and HRR deficiency (NCT04666740). The Southwest Oncology Group is conducting a study evaluating pembrolizumab with or without olaparib as a maintenance therapy in patients with mPDAC and germline BRCA1/2 mutations (NCT04548752).

Another question is about the relationship between diabetes and PDAC. Type 2 diabetes is a risk factor for the development of pancreatic cancer and, inversely, PDAC is associated with suspected causes of diabetes. There is progressive β-cell damage with the aberrant activation, and the depletion of the β-cell mass can induce both apoptosis and senescence. The insulin released from the β-cells is released into the portal circulation, which supplies the acinar and ductal cells adjacent to the islets. Both types of cells may be near to the islets and may be supplied from the portal circulation within the pancreas. This proximity allows high concentrations of pancreatic islet hormones to directly reach accumulations of the acinar and ductal cells, and attack the insulin receptors present on acinar cells and the IGF-I receptors on the transformed cells; this process affects both the survival and the spread [93].

## 5. Conclusions

It is necessary to evaluate novel targeted therapies in the treatment of pancreatic cancer by identifying its underlying pathological features, and including said features in the molecular biomarker studies. In the near future, the robust analysis of many genes and different mutations may help predict patient selection and pancreatic cancer treatment efficacy. This approach will hopefully identify the subsets of patients with specific biological behaviors and those who have increased responses to the treatment. This is an important step towards “personalized medicine” for pancreatic cancer patients.

## Figures and Tables

**Table 1:** Pathways overexpression in pancreatic cancer.

Pathway	Target	Incidence	Ref.
Angiogenesis	VEGFR, RON, c-Met, c-KIT, FLT-3, PDGF	90%	[22,23,24,25,26,27,28,29,30]
EGFR	EGFR	30–95%	[31,32,33,34,35,36,37]
IGF1R	IGF1R	60%	[38,39]
RAS	RAS/RAF/MEK/ERK	90–95%	[40,41,42]
PI3K/AKT/mTOR	PI3K/AKT/mTOR	55–60%	[43,44,45,46,47]
SRC	C-SRC	70%	[48,49,50]
JAK/STAT	JAK1, JAK2, JAK3, Tyk2, Stat3	-	[51,52,53,54,55]
NOTCH	NOTCH2/3R	-	[56,57,58,59,60]
HEDGEHOG	HH ligands	-	[61,62,63,64,65,66]
WNT	FZD1,2,5,7,8 R	-	[67,68,69,70]
TGFβ	TGFβ	-	[71,72,73]
ADP-RIBOSE	BRCA1/2	5–7%	[74,75,76,77,78,79,80]
